# Functional Analysis of Promoters from Three Subtypes of the PI3K Family and Their Roles in the Regulation of Lipid Metabolism by Insulin in Yellow Catfish *Pelteobagrus fulvidraco*

**DOI:** 10.3390/ijms19010265

**Published:** 2018-01-16

**Authors:** Mei-Qin Zhuo, Zhi Luo, Yi-Huan Xu, Dan-Dan Li, Ya-Xiong Pan, Kun Wu

**Affiliations:** 1 Freshwater Aquaculture Collaborative Innovative Centre of Hubei Province, Fishery College, Huazhong Agricultural University, Wuhan 430070, China; zhuomeiqin@webmail.hzau.edu.cn (M.-Q.Z.); xuyihuan@webmail.hzau.edu.cn (Y.-H.X.); ldd13007106896@webmail.hzau.edu.cn (D.-D.L.); biopyx@webmail.hzau.edu.cn (Y.-X.P.); pervcy@webmail.hzau.edu.cn (K.W.); 2 Laboratory for Marine Fisheries Science and Food Production Processes, Qingdao National Laboratory for Marine Science and Technology, Qingdao 266237, China; 3 Collaborative Innovation Center for Efficient and Health Production of Fisheries, Hunan University of Arts and Science, Changde 415000, China

**Keywords:** phosphatidylinositol-3 kinase, functional analysis of promoters, insulin, lipid metabolism, fish

## Abstract

In the present study, the length of 360, 1848 and 367 bp sequences of promoters from three subtypes of PI3K family (PI3KCa, PI3KC2b and PI3KC3) of yellow catfish *Pelteobagrus fulvidraco* were cloned and characterized. Bioinformatics analysis revealed that *PI3KCa*, *PI3KC2b* and *PI3KC3* had different structures in their core promoter regions. The promoter regions of *PI3KCa* and *PI3KC2b* had CpG islands but no CAAT and TATA box. In contrast, the promoter of *PI3KC3* had the canonical TATA and CAAT box but no CpG island. The binding sites of several transcription factors, such as HNF1, STAT and NF-κB, were predicted on *PI3KCa* promoter. The binding sites of transcription factors, such as FOXO1, PPAR-RXR, STAT, IK1, HNF6 and HNF3, were predicted on *PI3KC2b* promoter and the binding sites of FOXO1 and STAT transcription factors were predicted on *PI3KC3* promoter. Deletion analysis indicated that these transcriptional factors were the potential regulators to mediate the activities of their promoters. Subsequent mutation analysis and electrophoretic mobility-shift assay (EMSA) demonstrated that HNF1 and IK1 directly bound with *PI3KCa* and *PI3KC2b* promoters and negatively regulated the activities of *PI3KCa* and *PI3KC2b* promoters, respectively. Conversely, FOXO1 directly bound with the *PI3KC2b* and *PI3KC3* promoters and positively regulated their promoter activities. In addition, AS1842856 (AS, a potential FOXO1 inhibitor) incubation significantly reduced the relative luciferase activities of several plasmids of *PI3KC2b* and *PI3KC3* but did not significantly influence the relative luciferase activities of the *PI3KCa* plasmids. Moreover, by using primary hepatocytes from yellow catfish, AS incubation significantly down-regulated the mRNA levels of *PI3KCa*, *PI3KC2b* and *PI3KC3* and reduced triacylglyceride (TG) accumulation and insulin-induced TG accumulation, as well as the activities and the mRNA levels of several genes involved in lipid metabolism. Thus, the present study offers new insights into the mechanisms for transcriptional regulation of PI3Ks and for PI3Ks-mediated regulation of lipid metabolism by insulin in fish.

## 1. Introduction

Phosphatidylinositol-3 kinase (PI3K) is an intracellular transducer with lipid substrate specificity and implicates a wide range of signaling pathways involved in cell survival, proliferation and nutrient metabolism [1]. In mammals, eight members of PI3K family are obtained and they are termed as PI3KCa, PI3KCb, PI3KCd, PI3KCg, PI3KC2a, PI3KC2b, PI3KC2g and PI3KC3, respectively [2]. Recently, we cloned and characterized seven PI3K members (without *PI3KC2g*) from yellow catfish *Pelteobagrus fulvidraco* [3]. However, the underlying transcriptional mechanism and the function of each PI3K member were still unknown. The regulation of gene expression can occur at different steps ranging from DNA–RNA transcription to post-translational modification of protein [4]. It involves the interaction of transcription factors with the transcription machinery as well as changes in DNA structure (epigenetic process including CpG dinucleotide methylation) which influences accessibility of promoter sequences [5]. At present, studies on the functions of PI3Ks promoter are scarce. In mammals, several transcriptional factors—such as FOXO1, NF-κB and HIF—positively regulate *PI3KCa* transcription [6,7,8], while p53 and IK1 negatively regulate transcription of *PI3KCa* and *PI3KC2b* [9,10,11]. Studies also suggested that the transcription factor STAT, which can bind with the region of PI3K promoters, differed between fish and mammals [12,13]. However, to our best knowledge, information involved in the transcriptional regulation of PI3K members was very scarce in fish.

The members of PI3K family have a pivotal role in the metabolic action of insulin [14] and they mediated the regulation of lipid metabolism by insulin in yellow catfish, a widely distributed freshwater omnivorous fish [15]. FOXO1 is the downstream effectors of the PI3K-mediated signaling pathway and insulin suppresses FOXO1 via phosphorylation-dependent nuclear exclusion [16,17,18]. In addition, FOXO1 is also an important transcriptional factor which positively regulates PI3K transcription [7]. AS1842856 (AS), a selective FOXO1 antagonist, can inhibit the binding of FOXO1 to its target DNA and its transactivation [19]. Thus, it will offer new insights into the mechanisms for PI3Ks-mediated regulation of lipid metabolism by insulin via targeting FOXO1 binding sites in fish.

Lipid metabolism is regulated by lipogenesis, lipolysis and lipid transport and many crucial enzymes and transcription factors are involved in these processes. Enzymes related to lipid metabolism include the lipogenic enzymes (such as glucose 6-phosphate dehydrogenase (G6PD), 6-phosphogluconate dehydrogenase (6PGD), isocitrate dehydrogenase (ICDH), malic enzyme (ME) and FA synthase (FAS)) and the lipolytic enzymes (such as adipose triacylglyceride lipase (ATGL) and carnitine palmitoyltransferase 1 (CPT 1), acetylCoA carboxylase b (ACCb)) [20]. Several key transcriptional factors such as peroxisome proliferators-activated receptors—(PPARα) and (PPARγ)—involved the regulation of lipid homeostasis via lipolysis and lipogenesis [21,22,23]. Some transmembrane fatty acid transporter proteins, such as transmembrane fatty acid transporters (CD36), lipoprotein lipase (LPL) and the fatty acid binding protein (FABP1), also play pivotal roles in regulating lipid content in fish [24]. In order to study the underlying mechanism of PI3K-mediated regulation of lipid metabolism by insulin, it will be very important and meaningful to assay the changes of these related enzymes activities and genes expressions following the insulin and AS treatment.

Yellow catfish *Pelteobagrus fulvidraco*, an omnivorous freshwater fish, is widely distributed in the inland freshwater waters in several Asian countries including China. The fish is regarded as a good candidate for freshwater culture in China for its delicious meat and high market value. Recently, under intensive aquaculture for this fish species, excess lipid deposition in yellow catfish become more and more widespread, which greatly reduce its taste and economic value. Thus, it is very important to investigate the characteristics of lipid metabolism and explore the pathway for reducing lipid deposition in yellow catfish. PI3Ks implicate a wide range of signaling pathways involved in lipid metabolism. Thus, as a part of our ongoing research into the functional analysis of PI3K members and the mechanism of PI3Ks-mediated regulation of lipid metabolism by insulin in yellow catfish, we identified and characterized the upstream 5′-flanking region of three subtypes of PI3K members (*PI3KCa*, *PI3KC2b* and *PI3KC3*) from yellow catfish. Several transcription factors binding sites of their promoters from *PI3KCa*, *PI3KC2b* and *PI3KC3* were demonstrated by using deletion, mutation and electrophoretic mobility-shift assay (EMSA). We confirmed that PI3Ks were regulated by FOXO1 at the transcriptional level and strong inhibition of FOXO1 binding sites led to transcriptional inactivation of PI3Ks, which in turn impaired insulin-induced lipogenesis in hepatocytes from yellow catfish. Meantime, targeting FOXO1 with AS not only reduced lipogenesis but also lipolysis and lipid transport in hepatocytes from yellow catfish.

## 2. Results

### 2.1. Cloning and Sequences Analysis of Promoters of PI3KCa, PI3KC2b and PI3KC3

**Figure 1:**
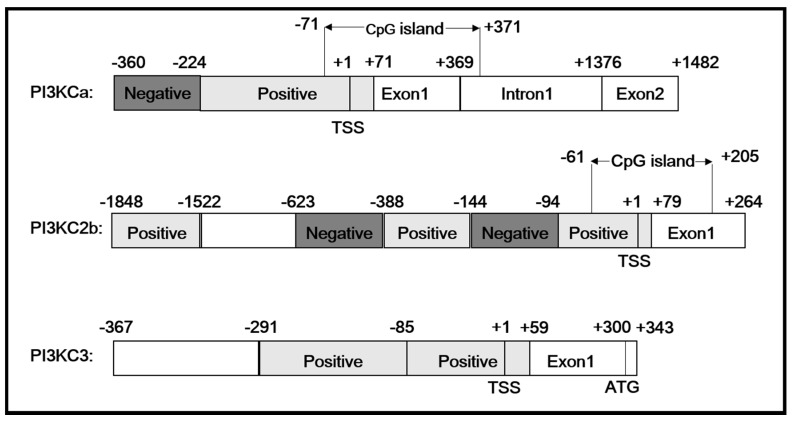
The schematic diagram of *PI3KCa*, *PI3KC2b* and *PI3KC3* gene structure. Positive: the region that positively regulated the promoter activity. Negative: the region that negatively regulated the promoter activity. TSS: transcription start site. ATG: translation initiation site. CpG island: the region that rich of Cytosine nucleotide (C) and Guanine nucleotide (G).

**Figure 2:**
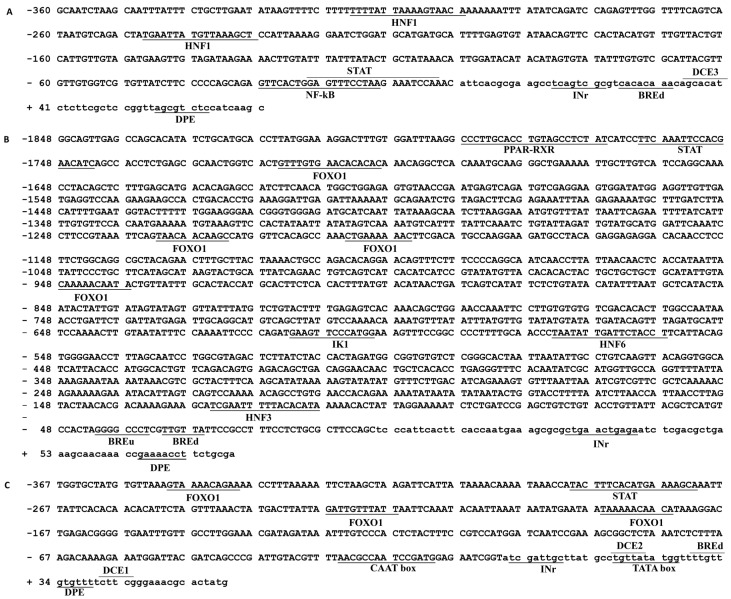
Nucleotide sequence of the 5′-flanking region of the *PI3KCa* (**A**), *PI3KC2b* (**B**) and *PI3KC3* (**C**) gene. Numbers are relative to the transcription start site (+1). The upstream sequences of transcription start site are in the capital letters, while the downstream sequences of transcription start site are in lowercase letters. The putative transcription factor binding sites are underlined.

**Figure 3:**
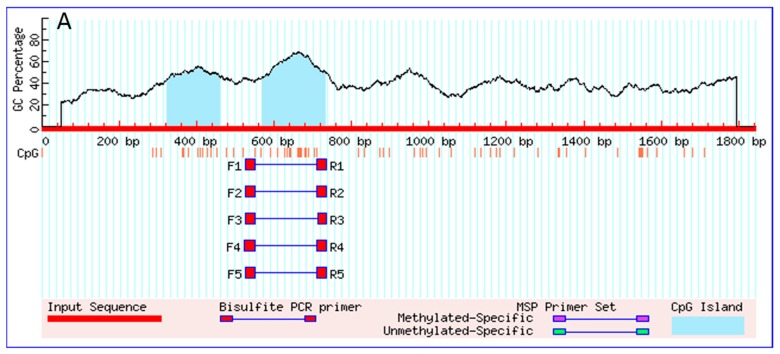

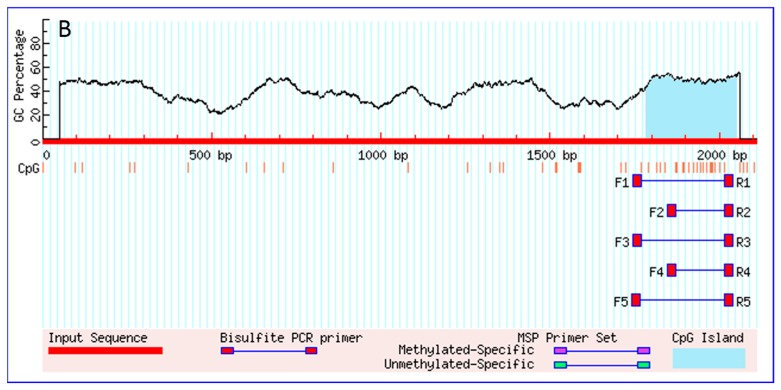
The prediction results of CpG islands in *PI3KCa* (**A**) and *PI3KC2b* (**B**) gene. The blue area indicates the region of CpG Island.

The transcription start sites (TSS) of *PI3KCa*, *PI3KC2b* and *PI3KC3* were identified according to our previous studies [3] and the first nucleotide of 5′cDNA of *PI3KCa*, *PI3KC2b* and *PI3KC3* was designated as +1. An intron with 1048 bp sequences was cloned at the position of +370 bp of *PI3KCa* and no introns were found within +264 bp of *PI3KC2b* and +343 bp of *PI3KC3* (Figure 1). The 360, 1848 and 367 bp sequences of promoters of *PI3KCa* (GenBank accession no.: MG574318), *PI3KC2b* (GenBank accession no.: MG574319) and *PI3KC3* (GenBank accession no.: MG574320) were cloned by using hiTAIL-PCR, respectively (Figure 2). A cluster of putative binding sites of several transcription factors such as HNF, STAT and NF-κB were predicted on *PI3KCa* promoter (Figure 2A) and the binding sites of transcription factors such as FOXO1, PPAR-RXR, STAT, IK1 and HNF were predicted on *PI3KC2b* promoter (Figure 2B) and the binding sites of FOXO1 and STAT transcription factors were predicted on *PI3KC3* promoter (Figure 2C). A TATA box and CAAT box located at +18 bp and −25 bp in the core promoter of *PI3KC3* were predicted (Figure 2C). Several conserved elements around the TSS, such as B recognition element (BRE), downstream core element (DCE) and DNA recognition element (DRE), were predicted in *PI3KCa*, *PI3KC2b* and *PI3KC3* (Figure 2). Furthermore, CpG islands in the *PI3KCa* and *PI3KC2b* promoter region and exon 1 were characterized by using the NCBI genome database and the online tools of Methprimer (Figure 3).

### 2.2. 5′-Deletion Assay of the PI3KCa, PI3KC2b and PI3KC3 Promoter

**Figure 4:**
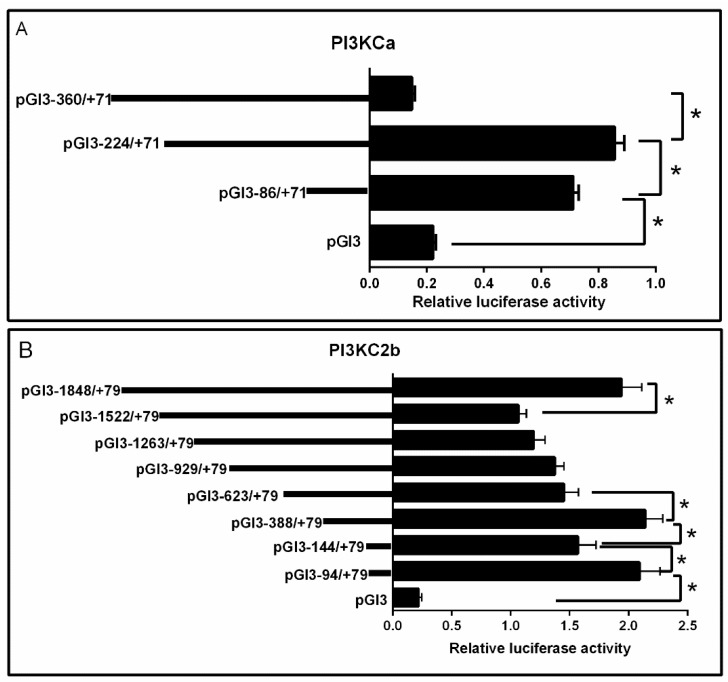

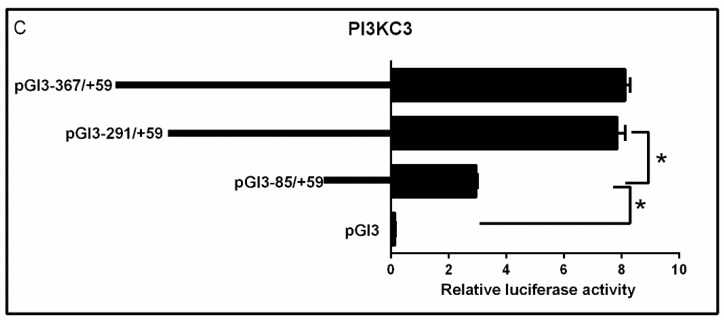
5′ unidirectional deletion analysis of the *PI3KCa*, *PI3KC2b* and *PI3KC3* promoter region for yellow catfish. Schematic diagram of truncated promoters are shown at left panel. The corresponding luciferase reporter assay results are shown at right panel. (**A**) A series of plasmids containing 5′ unidirectional deletions of the *PI3KCa* promoter region (pGl3-360, −224, −86 and pGl3-basic) fused in frame to the luciferase gene were transfected into HepG2 cells; (**B**) A series of plasmids containing 5′ unidirectional deletions of the *PI3KC2b* promoter region (pGl3-1848, −1522, −1263, −929, −623, −388, −144, −94 and pGl3-basic) fused in frame to the luciferase gene were transfected into HepG2 cells; (**C**) A series of plasmids containing 5′ unidirectional deletions of the *PI3KCa* promoter region (pGl3-367, −291, −85 and pGl3-basic) fused in frame to the luciferase gene were transfected into HepG2 cells. Values represent the ratio between firefly and Renilla luciferase activities. Results are expressed as the mean ± SEM arbitrary units of three independent experiments. Symbol (*) indicates significant differences between the two group (*p* < 0.05).

Deletion of the sequences from −360 to −224 bp of *PI3KCa* significantly increased the relative luciferase activity. Subsequent deletion to −86 bp significantly decreased the relative luciferase activity (Figure 4A).

Deletion of the sequences from −1848 to −1522 bp of *PI3KC2b* significantly decreased the relative luciferase activity and there is no significant difference after the deletion of the sequences from −1522 to −623 bp. The deletion of the sequences from −623 to −388 bp significantly enhanced the relative luciferase activity and truncation to −144 bp significantly reduced the relative luciferase activity and the relative luciferase activity significantly increased after subsequent deletion of the sequences from −144 to −94 bp of *PI3KC2b* (Figure 4B).

Deletion of the sequences from −367 to −291 bp of *PI3KC3* showed no significant difference on the relative luciferase activity and subsequent deletion to −85 bp significantly decreased the relative luciferase activity (Figure 4C).

### 2.3. Site-Mutation Analysis

**Figure 5:**
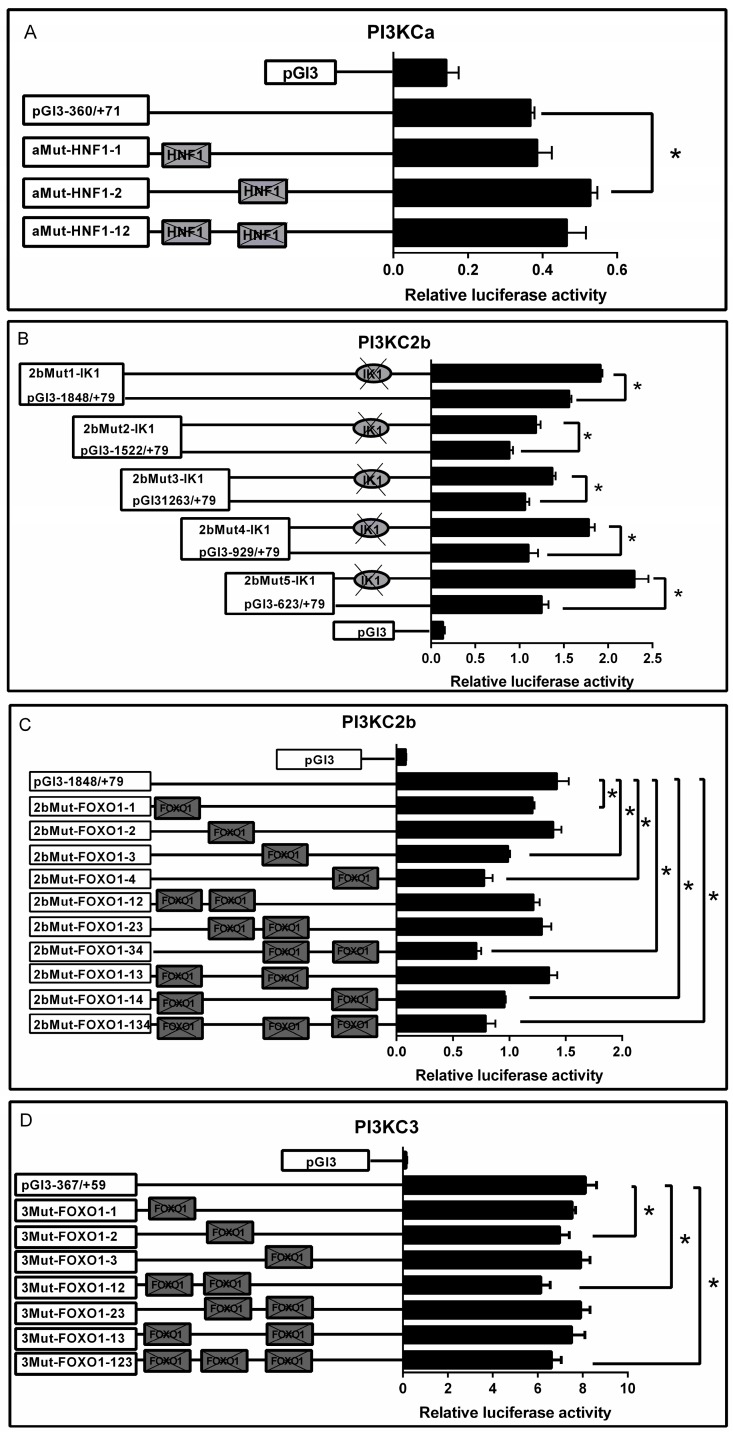
Analysis of putative transcript factor binding sites by site-directed mutagenesis. (**A**) Site-mutations of HNF1 binding sites on pGl3-360/+71 of *PI3KCa* vector; (**B**) Site-mutation of IK1 binding site on pGl3-1848/+79, pGl3-1522/+79, pGl3-1263/+79, pGl3-929/+79 and pGl3-623/+79 of *PI3KC2b* vectors; (**C**) Site-mutation of FOXO1 binding sites on pGl3-1848/+79 of *PI3KC2b* vector; (**D**) Site-mutation of FOXO1 binding sites on pGl3-367/+59 of *PI3KC3* vector. Values represent the ratio between firefly and Renilla luciferase activities. Results are expressed as the mean ± SEM arbitrary units of three independent experiments. Symbol (*) indicates significant differences between the two group (*p* < 0.05).

Compared with the wild type pGl3-360/+71 of *PI3KCa* vectors, the mutation of −304/−300 (aMut-HNF1-1) HNF1 binding site did not change the relative luciferase activity, while the mutation of −238/−236 (aMut-HNF1-2) HNF1 binding site significantly increased the relative luciferase activity. However, there is no significant difference after the co-mutation of ((−304/−300) + (−238/−236)) (aMut-HNF1-12) HNF1 binding sites compared with the wild type pGl3-360/+71 of *PI3KCa* vectors. (Figure 5A).

The mutation of −609/−605 IK1 binding sites (2bMut1-IK1, 2bMut2-IK1, 2bMut3-IK1 and 2b-Mut4-IK1) significantly increased the luciferase activity when compared with their corresponding wild type *PI3KC2b* vectors (Figure 5B). Compared with the wild type pGl3-1848/+79 of *PI3KC2b* vectors, the relative luciferase activities were significantly reduced after the mutation of −1708/−1705 (2bMut-FOXO1-1), −1202/−1198 (2bMut-FOXO1-3) and −947/−943 (2bMut-FOXO1-4) FOXO1 binding sites but showed no significant changes after the mutation of −1231/−1227 (2bMut-FOXO1-2) FOXO1 binding sites. The co-mutation of ((−1708/−1705) + (−947/−943)) (2bMut-FOXO1-14) and ((−1202/−1198) + (−947/−943)) (2bMut-FOXO1-34) FOXO1 binding sites significantly reduced the relative luciferase activity, while the co-mutation of ((−1708/−1705) + (−1231/−1227)) (2bMut-FOXO1-12), ((−1231/−1227) + (−1201/−1198)) (2bMut-FOXO1-23) and ((−1708/−1705) + (−1202/−1198)) (2bMut-FOXO1-13) FOXO1 binding sites showed no significant difference in the relative luciferase activity. In addition, compared with the wild type pGl3-1848/+79 of *PI3KC2b* vectors, the relative luciferase activity was significantly reduced after the co-mutation of ((−1708/−1705) + (−1231/−1227) + (−1201/−1198)) (2bMut-FOXO1-134) FOXO1 binding sites (Figure 5C).

For site-mutation analysis of *PI3KC3* promoter, compared with the wild type pGl3-367/+59 of *PI3KC3* vectors, the relative luciferase activities were significantly reduced after the mutation of −224/−221 (3Mut-FOXO1-2) FOXO1 binding site and showed no significant difference after the mutation of −347/−344 (3Mut-FOXO1-1) and −183/−179 (3Mut-FOXO1-3) FOXO1 binding sites. Compared with the wild type pGl3-367/+59 of *PI3KC3* vectors, the co-mutation of ((−347/−344) + (−224/−221)) (3Mut-FOXO1-12) and ((−347/−344) + (−224/−221) + (−183/−179)) (3Mut-FOXO1-123) FOXO1 binding sites significantly decreased the relative luciferase activities, whereas there are no significant changes after the co-mutation of ((−347/−344) + (−183/−179)) (3Mut-FOXO1-13) and ((−224/−221) + (−183/−179)) (3Mut-FOXO1-23) FOXO1 binding sites (Figure 5D).

### 2.4. HNF1, FOXO1 and IK1 Bind with the Promoters

**Figure 6:**
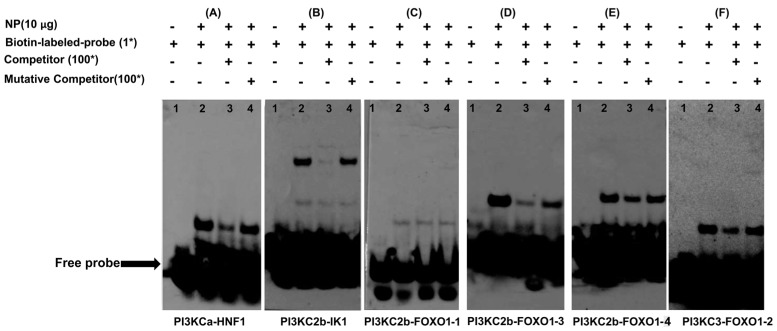
Electrophoretic mobility-shift assay (EMSA) of putative transcription factors binding sequence. The 5′-biotin labeled double-stranded oligomers were incubated with HepG2 nuclear protein (NP) extract. A 100-fold excess of the competitor and mutative competitor oligomers was added to the competition and mutant competition assay, respectively. The oligonucleotide sequences are given in Supplementary Table S4. (**A**) HNF1 binding sequences located at −249/−228 bp of *PI3KCa* promoter; (**B**) FOXO1 binding sequences located at −1719/−1698 bp of *PI3KC2b* promoter; (**C**) FOXO1 binding sequences located at −1211/−1190 bp of PI3KC2b promoter; (**D**) FOXO1 binding sequences located at −952/−931 bp of *PI3KC2b* promoter; (**E**) IK1 binding sequences located at −619/−597 bp of *PI3KC2b* promoter; (**F**) FOXO1 binding sequences located at −232/−211 bp of *PI3KC3* promoter. The symbols “+” or “−” in the top row indicate the presence or absence of nuclear protein extract, probes, competitors and mutative competitors.

According to the results of site-mutation analysis, we speculated that HNF1 bound with the −238/−236 site on *PI3KCa* promoter, IK1 bound with the −609/−605 site on *PI3KC2b* promoter and FOXO1 bound with the −1708/−1705, −947/−943 and −1202/−1198 sites on *PI3KC2b* promoter and FOXO1 bound with −224/−221 site on *PI3KC3* promoter. Thus, we next examined their ability to physically interact with HNF1, FOXO1 and IK1 by using the EMSA assay. The 100-fold unlabeled HNF1 binding sequence (located at −249 to −228 bp of *PI3KCa* promoter) competed for binding when HNF1 binding sequence was used as probe, while the 100-fold unlabeled Mut-HNF1 binding sequence markedly reduced this competition, indicating that this sequence was bound by HNF1 factor (Figure 6A). Similar results were also found for IK1 binding sequence located at −619/−597 bp of *PI3KC2b* promoter (Figure 6B). However, no band was found when using FOXO1 binding sequence (located at −1719/−1698 bp of *PI3KC2b* promoter) as probe, suggesting that this sequence was not bound by any factors (Figure 6C). In addition, we also found that the 100-fold unlabeled FOXO1 binding sequence located at −1212/−1190 bp of *PI3KC2b* promoter competed for binding when FOXO1 binding sequence was used as probe, while the 100-fold unlabeled Mut-FOXO1 binding sequence reduced this competition, indicating that this sequence was bound by FOXO1 factor (Figure 6D) and the similar results were also found for FOXO1 binding sequence located at −952/−931 bp of *PI3KC2b* promoter(Figure 6E) and −232/−211 bp of *PI3KC3* promoter (Figure 6F).

### 2.5. Effect of AS on Plasmid Luciferase Activity

**Figure 7:**
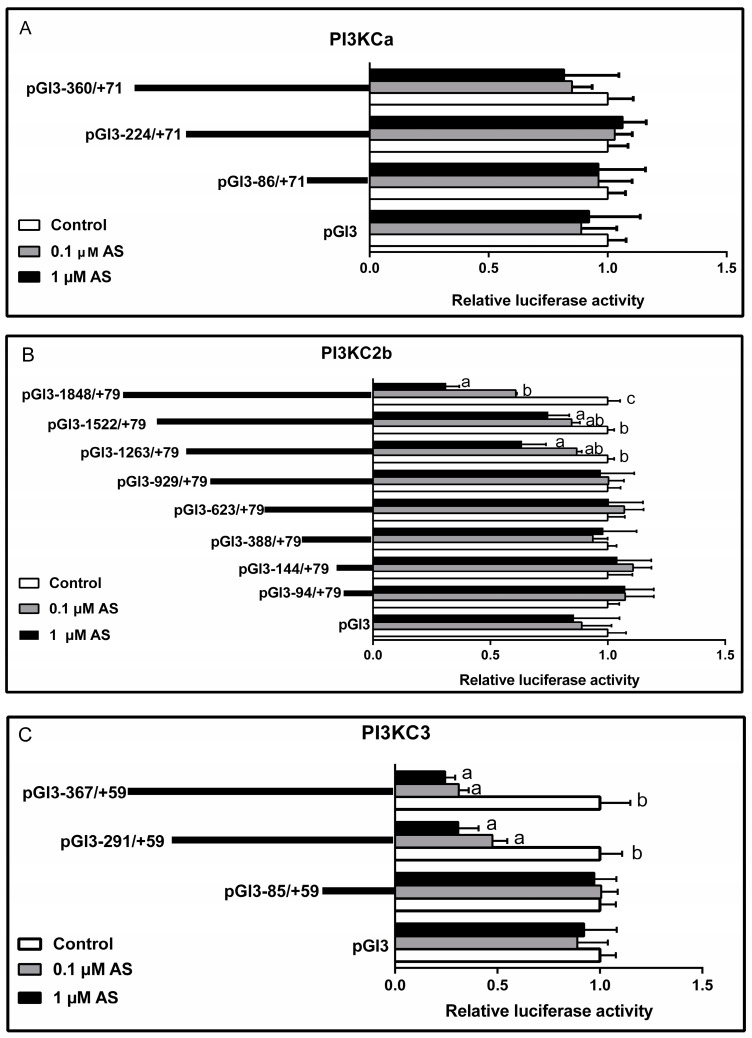
(**A**) Effects of AS on the promoter activity of different *PI3KCa* promoter plasmid in HepG2 cells; (**B**) Effects of AS on the promoter activity of different *PI3KC2b* promoter plasmid in HepG2 cells; (**C**) Effects of AS on the promoter activity of different *PI3KC3* promoter plasmid in HepG2 cells. The promoter activity of each plasmid without AS treatment was consider as relative luciferase activity 1. Bars that do not share a common letter (a, b, c) mean significant difference among three treatments (*p* < 0.05).

AS incubation showed no significant effects on the relative luciferase activity of all *PI3KCa* vectors (Figure 7A). The relative luciferase activities of the pGl3-1848/+79, pGl3-1522/+79 and pGl3-1263/+79 of *PI3KC2b* vectors were significantly reduced after 1 μM AS incubation, while the relative luciferase activities of other *PI3KC2b* vectors were relatively stable (Figure 7B). In addition, both 1 μM and 0.1 μM AS incubation significantly down-regulated the relative luciferase activity of pGl3-367/+59 and pGl3-291/+59 of *PI3KC3* vectors but showed no significant effects on pGl3-85/+59 of *PI3KC3* vectors (Figure 7C).

### 2.6. Effect of AS on PI3Ks Gene Expressions

**Figure 8:**
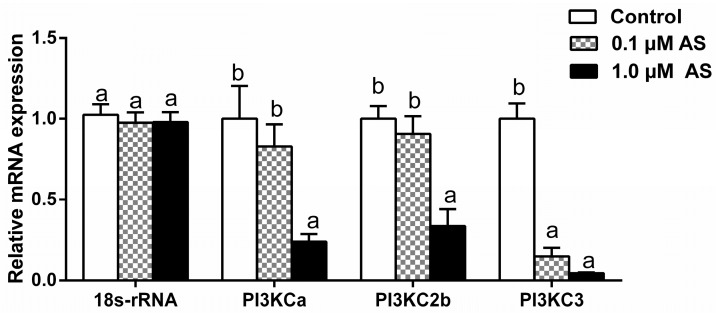
Effects of AS on the mRNA expression of PI3Ks in primary hepatocytes from yellow catfish. Values are expressed as means + SEM (*N* = 3 independent biological experiments). Bars that do not share a common letter (a, b) mean significant difference among three treatments (*p* < 0.05).

The mRNA levels of *PI3KCa*, *PI3KC2b* and *PI3KC3* were significantly reduced after incubation with 1 μM AS at concentration. 0.1 μM AS treatment significantly decreased the mRNA levels of *PI3KC3* but showed no effects on the *PI3KCa* and *PI3KC2b* expressions (Figure 8).

### 2.7. Effect of Insulin and/or AS on TG Accumulation, Enzymatic Activity and Gene Expressions

**Figure 9:**
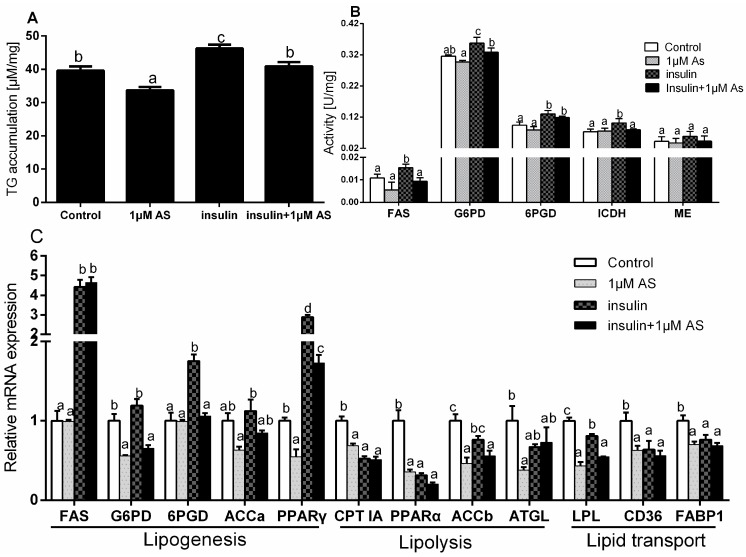
Effects of insulin and/or AS on TG accumulation (**A**), the enzyme activities (**B**) and the mRNA expression of several genes involved in lipid metabolism (**C**) in primary hepatocytes from yellow catfish. Values are expressed as means + SEM (*N* = 3 independent biological experiments). Bars that do not share a common letter (a, b, c, d) mean significant difference among the treatments (*p* < 0.05).

Compared with the control, 1 μM AS alone significantly reduced TG accumulation. Compared with single insulin incubation, pre-treatment with 1 μM AS also significantly decreased insulin-induced TG accumulation (Figure 9A).

Compared with the control, single AS treatment did not significantly influence the activities of FAS, G6PD, 6PGD, ICDH and ME. Compared with insulin treatment alone, AS pre-treatment significantly reduced insulin-induced up-regulation of the activities of FAS, G6PD and ICDH but showed no significant effects on 6PGD and ME activities (Figure 9B). 

Compared with the control, AS alone showed no significant effects on the mRNA levels of *FAS*, *6PGD* and *ACCa* but down-regulated the mRNA expression of *G6PD*, *PPARγ*, *PPARα*, *CPT IA*, *ACCb*, *ATGL*, *LPL*, *CD36* and *FABP1*. Insulin treatment significantly up-regulated the mRNA expression of *FAS*, *6PGD* and *PPARγ* but down-regulated the mRNA levels of *CPT IA*, *PPARα*, *LPL*, *CD36* and *FABP1* and showed no effects on the *G6PD*, *ACCa* and *ACCb* expressions. Compared with insulin treatment alone, AS pre-treatment significantly reduced the insulin-induced up-regulation of *G6PD*, *6PGD* and *PPARγ* expressions. Moreover, AS pre-treatment also significantly increased the insulin-induced down-regulation of *ACCb* and *LPL* expressions (Figure 9C).

## 3. Discussion

Although the characterization and tissue expression profile of seven PI3K members from yellow catfish were determined in our previous study [3], the underlying transcriptional mechanisms of PI3K member were still unknown. As a continuation of our series of studies involved in the structure and functional analysis of PI3K in yellow catfish, the present study cloned and characterized the sequences of PI3Ks promoter and explored theirs functions. Unfortunately, only the sequence of *PI3KCa*, *PI3KC2b* and *PI3KC3* promoters were cloned, despite our enormous efforts. Recently, we also found that the block of PI3K (using Wortmannin) at phosphorylation level influenced insulin-induced changes of lipid metabolism in yellow catfish [15]. However, the effect of insulin on lipid metabolism in yellow catfish after the block of PI3K at transcriptional level remains unknown. Thus, exploring the mechanism of insulin-induced changes of lipid metabolism at transcriptional level may play new insights into the regulation of lipid metabolism by insulin. 

In eukaryotes, the term “core promoter” is often used to focus on the DNA region in the immediate vicinity of the TSS, which is assumed to dock the pre-initiation complex [5]. In the present study, the core promoters of *PI3KCa*, *PI3KC2b* and *PI3KC3* had different structures. *PI3KCa* and *PI3KC2b* had CpG islands but no CAAT and TATA box, while *PI3KC3* had the canonical TATA and CAAT box but no CpG island. Moreover, the present study indicated that the TATA box and CAAT box were located at +18 bp and −25 bp of *PI3KC3* promoter, respectively, which were distinguished from other report that TATA box was always located at the 5′ upstream region [25]. Interestingly, we also found that the average activities of *PI3KCa*, *PI3KC2b* and *PI3KC3* promoters were approximately 2, 7 and 45-fold of pGl3-basic empty vector activity, respectively. Thus, we speculated that GC-rich and TATA-less regions probable negatively regulated promoter activity. Bird [26] suggested that methylation of CpG islands within gene promoters was generally thought to silence gene expression. In addition, several common transcription initiation elements, such as BRE, INR, DCE and DRE [6], also existed in the core promoters of *PI3KCa*, *PI3KC2b* and *PI3KC3* from yellow catfish, which were the basic elements in driving gene transcription. 

Identification of transcription factor binding site (TFBS) plays an important role in deciphering the mechanisms of gene regulation [27]. The present study identified that the binding sites of STATs were located at the position of −22/−3 bp of *PI3KCa* promoter, −1761/−1743 bp of *PI3KC2b* promoter and −290/−270 bp of *PI3KC3* promoter. By using deletion analysis, we also found that the regions from −224/+71 bp of *PI3KCa* promoter, −1845 to −1522 bp of *PI3KC2b* promoter and −291 to −85 bp of *PI3KC3* promoter positively regulated their corresponding promoter activity, indicating that STAT was a potential positive regulator which mediated PI3K promoter activity from yellow catfish. STAT is a latent cytoplasmic transcription factor that participates in gene regulation and plays important roles in multiple biological processes [12,13]. Similarly, Abell et al. [28] also reported that STAT positively regulated the expression of PI3K regulatory subunit in mammary gland tissue. NF-κB is a dimeric transcription factor and exists in all cell types and plays pivotal roles in inflammatory processes, angiogenesis, immunity and apoptosis [29,30]. The present study also found a NF-κB binding site near the STAT binding site on *PI3KCa* promoter, enlightening us that there may be an interaction between STAT and NF-κB in PI3KCa-mediated transcriptional activation. In human, Yang et al. [8] confirmed that *PI3KCa* was transcriptionally regulated through NF-κB pathway. PPARs are key transcriptional factors which mediate the regulation of many enzymes in lipid metabolism [31,32]. In the present study, we identify that the PPAR-RXR binding site (located at −1788/−1767 bp of *PI3KC2b* promoter) is a potential positive regulator in regulating *PI3KC2b* promoter activity from yellow catfish. In mammals, studies also suggested that PPAR-RXR positively regulated the expression of *PI3K* regulatory subunit [33,34,35].

In the present study, the deletion analysis indicated that the region from −360 to −224 bp of *PI3KCa* promoter negatively regulated *PI3KCa* promoter activity and the region from 623 to −388 bp and −144 to −94 bp of *PI3KC2b* promoter also negatively regulated *PI3KC2b* promoter activity. Interestingly, two HNF1 binding sites were predicted within the region from −360 to −224 bp of *PI3KCa* promoter and a HNF6 binding site was predicted within the region from −623 to −388 bp of *PI3KC2b* promoter and a HNF3 binding site was predicted within −144 to −94 bp of *PI3KC2b* promoter, indicating that HNFs were the potential negative regulators for PI3K promoter activity in yellow catfish. Moreover, the site-directed mutagenesis and EMSA analysis confirmed that the HNF1 factors directly bound with the sequence from the −249 to −227 bp of *PI3KCa* promoter and negatively regulated promoter activity. HNF1 is a liver-enriched transcription factor and it plays a key role in regulating liver-specific gene expression [36]. Studies have shown that HNF1 bound as homodimers to a small set of liver-enriched transcription factors (such as C/EBP, HNF-3 and HNF-4), thereby modulating the expression of liver-enriched genes [36]. In addition, it seemed that a negative correlation existed between expression of *HNF1* and *PI3KCa*. Similarly, Li [37] suggested that mRNA expression of *HNF1* was notably decreased in ovarian cancer tissues while *PI3KCa* expression was high in ovarian cancer tissues [37,38]. Meantime, an IK1 binding site located within the region from −623 to −388 bp of *PI3KC2b* promoter was predicted. IK1 encodes a DNA-binding zinc finger protein and regulates expression of genes involved in important biological pathways [11]. In mammals, IK1 was a central regulator of hematopoiesis and is required lymphocyte development [39]. Interestingly, PI3K-C2b also plays a key role in the activation and the proliferation of lymphocyte [40]. Recently, studies have suggested that *PI3KC2b* was one of the target genes of IK1 in leukemia [11]. In this study, we discovered that mutation on the −609/−605 IK1 binding site significantly reduced *PI3KC2b* promoter activity. Moreover, EMSA assay confirmed that the sequence at −615 to −593 bp of *PI3KC2b* promoter was a functional IK1 binding locus.

FOXO1 plays a central role in the regulation of the metabolism, stress response and apoptosis [41] and it also integrates insulin signaling in regulating glucose and lipid metabolisms [42,43]. For example, FOXO1 acts in concert with PPARγ co-activator to stimulate lipid deposition [44,45]. However, very few studies was conducted on whether FOXO1 can integrate with PI3Ks in regulating lipid metabolism. AS, a selective FOXO1 antagonist, can inhibit the binding of FOXO1 to the target DNA [19]. In the present study, we found that the relative luciferase activities of the pGl3-1848/+79, pGl3-1522/+79 and pGl3-1263/+79 of *PI3KC2b* vectors and the pGl3-367/+59 and pGl3-291/+59 of *PI3KC3* vectors were significantly reduced after AS incubation. The site-directed mutagenesis assay indicated that the relative luciferase activities of *PI3KC2b* promoter significantly decreased after site-mutation on the −1708/−1705, −1202/−1198 and −947/−943 FOXO1 binding sites and the similar result was found for *PI3KC3* promoter after site-mutation on the −224/−221 FOXO1 binding sites. Furthermore, EMSA assay demonstrated that the −1202/−1198 and −947/−943 FOXO1 binding sites were the functional binding locus on *PI3KC2b* promoter and also the −224/−221 FOXO1 binding sites was a functional binding locus on *PI3KC3* promoter. Similarly, Hui et al. [7] reported that FOXO1 positively regulated the *PI3KCa* transcription. However, the present study found no FOXO1 binding site on the *PI3KCa* promoter, which was probably due to the short length of *PI3KCa* promoter obtained here.

After finding that FOXO1 positively regulated *PI3KC2b* and *PI3KC3* promoter activity, next we explored whether FOXO1 regulated the *PI3Ks* transcription in yellow catfish. Thus, AS was used to incubation the primary hepatocytes from yellow catfish. The results indicated that AS treatment significantly reduced the transcript levels of *PI3KCa*, *PI3KC2b* and *PI3KC3* in primary hepatocytes and *PI3KC3* was more sensitive to AS than *PI3KCa* and *PI3KC2b*. Thus, the present study, for the first time, demonstrated that *PI3KCa*, *PI3KC2b* and *PI3KC3* were regulated by FOXO1 at the transcriptional levels.

Our previous study indicated that insulin regulated lipid metabolism via PI3K pathway [15]. The mechanism about the mechanism of PI3K-mediated regulation of lipid metabolism by insulin was explored. The present study indicated that AS significantly reduced insulin-induced TG accumulation. Similarly, targeting FOXO1 with AS suppressed adipogenesis in adipocytes [44,45]. To investigate the underlying mechanism of AS reducing TG accumulation in hepatocytes, the enzyme activities and the mRNA expression of genes related to lipid metabolism were analyzed. G6PD, 6PGD, ICDH and ME play an important role in generating NAD(P)H, which are necessary for lipogenesis [20]. AS pre-treatment significantly reduced insulin-induced up-regulation of the activities of FAS, G6PD and ICDH, indicating that the reduction of the insulin-induced TG accumulation by AS could be attributable to the down-regulation of these lipogenesis enzymes. In general, hepatic lipid metabolism is maintained by the regulation of several genes involved in lipogenesis (such as *FAS*, *G6PD*, *6PGD*, *ACCa* and *PPARγ*), lipolysis (such as *ATGL*, *ACCb*, *PPARα* and *CPT IA*) and lipid transport (*LPL*, *CD36* and *FABP1*) [20]. In this study, AS pre-treatment significantly reduced the insulin-induced up-regulation of the mRNA level of *G6PD*, *6PGD* and *PPARγ*, suggesting that insulin regulated *G6PD*, *6PGD* and *PPARγ* gene expressions probably via FOXO1/PI3K pathway in yellow catfish. Similarly, Zou et al. [44] and Liu et al. [45] also found that AS decreased *PPARγ* expression in adipocytes. Interestingly, our results showed that the changes of FAS, G6PD and 6PGD activities did not parallel with their mRNA expressions following the AS treatment. Similarly, in our previous study, we also found that insulin changes FAS and G6PD activity but not changes their mRNA level after 24 h incubation on hepatocytes from yellow catfish [46]. Chen et al. [47] also suggested that Cu excess did not changes 6PGD activity but changes its mRNA levels in the liver from yellow catfish. As mentioned above, gene expression is affected by mRNA and protein stability and also time-course dependent. AS treatment alone also significantly down-regulated the expression of several lipolysis and lipid transport genes, such as *CPT1A*, *PPARα*, *ACCb*, *ATGL*, *CD36*, *FABP1* and *LPL*, indicating that AS not only reduced lipogenesis but also the action of lipolysis and lipid transport in yellow catfish. Similarly, several studies pointed out that the inhibition of FOXO1 also reduced the expression of *LPL* and *CD36* [48,49]. Moreover, studies also suggested that FOXO1 bound with the *ATGL* promoter and increased *ATGL* expression in adipocytes [50,51].

In summary, the length of 360, 1848 and 367 bp sequences of *PI3KCa*, *PI3KC2b* and *PI3KC3* promoters from yellow catfish were cloned and characterized. The different structure of core promoters of *PI3KCa*, *PI3KC2b* and *PI3KC3* was proposed. Several TFBS of *PI3KCa*, *PI3KC2b* and *PI3KC3* promoters were demonstrated. In addition, we confirmed that PI3Ks were transcriptionally regulated through FOXO1 pathway. Furthermore, PI3Ks mediated the insulin-induced changes of lipid metabolism by targeting FOXO1 and inhibition FOXO1 with AS not only reduced lipogenesis but also lipolysis and lipid transport in hepatocytes from yellow catfish.

## 4. Materials and Methods

### 4.1. Experimental Animals and Reagents

Yellow catfish were obtained from Hubei Bairong Fisheries Farm (Huanggang, Hubei Province, China). HepG2 cell lines were obtained from the Cell Resource Center in the Fishery College of Huazhong Agricultural University. Dulbecco’s Modified Eagles Medium (DMEM), 0.25% trypsin-EDTA and fetal bovine serum (FBS) were obtained from Gibco/Invitrogen, USA. AS1842856, dimethyl sulphoxide (DMSO), penicillin, streptomycin, trypan blue and other reagents were purchased from Sigma-Aldrich (St. Louis, MO, USA). We ensured that the experiments were performed in accordance with the experimental protocols of Huazhong Agricultural University (HZAU) and approved by the ethics committee of HZAU (identification code: Fish-2016-0419, Date: 19 April 2016)

### 4.2. Experimental Treatment

Two experiments were carried out. Exp. 1 was conducted to clone the promoter sequences of *PI3KCa*, *PI3KC2b* and *PI3KC3* from yellow catfish. Exp. 2 was conducted to determine their potential roles in insulin influencing lipid metabolism.

#### 4.2.1. Exp. 1: Cloning and Functional Analysis of *PI3KCa, PI3KC2b* and *PI3KC3* Promoters

##### Cloning of Promoters and 5′-Deletion Plasmids Construction

The 5′ cDNA sequences of *PI3KCa* (GenBank accession No. KU976455), *PI3KC2b* (GenBank accession No. KU976460) and *PI3KC3* (GenBank accession No. KU976461) from yellow catfish were obtained according to our previous study [3]. Genomic DNA was extracted from yellow catfish tail fins using a commercial kit (Omega, Norcross, GA, USA). A series of primers (Supplementary Table S1) were designed to determine the position of the first intron of *PI3KCa*, *PI3KC2b* and *PI3KC3*. The sequences of *PI3KCa*, *PI3KC2b* and *PI3KC3* promoters were cloned using the hiTAIL-PCR method [52]. The specific primers with overlapping sequence were listed in Supplementary Table S1. For the generation of the luciferase reporter constructs, the PCR products and pGl3-Basic vectors (Promega, Madison, WI, USA) were purified and digested using corresponding endonucleases and then products were ligated using ClonExpress^TM^ II One Step Cloning Kit (Vazyme, Piscataway, NJ, USA). The plasmids were named as pGl3-360/+71 of *PI3KCa* vector, pGl3-1848/+79 of *PI3KC2b* vector and pGl3-367/+59 of *PI3KC3* vector, respectively, according to the distance from their transcription start sites. Plasmids pGl3-224/+71, pGl3-86/+71 of *PI3KCa* vector, which contained unidirectional deletions of the promoter, were generated with the Erase-a-Base system (Promega) using templates of pGl3-360/+71 of *PI3KCa* vector. Similarly, plasmids pGl3-1522/+79, pGl3-1263/+79, pGl3-929/+79, pGl3-623/+79, pGl3-388/+79 and pGl3-144/+79 of *PI3KC2b* vector, as well as pGl3-291/+59 and pGl3-85/+59 of *PI3KC3* vectors, were generated with the Erase-a-Base system (Promega) using pGl3-1848/+79 of *PI3KC2b* vector and pGl3-367/+59 of *PI3KC3* vector as templates, respectively. The PCR reactions were performed using the TaKaRa PrimeSTAR^®^ HS DNA Polymerase kit (TaKaRa, Tokyo, Japan) under the following PCR conditions: 94 °C for 5 min, then 30 cycles of 94 °C for 15 s, 55 °C for 30 s and 72 °C for 1 min, followed by a final extension at 72 °C for 5 min. All plasmids were sequenced for verification in a commercial company (Tsingke, Wuhan, China). The primer sequences used for plasmid construction are shown in Supplementary Table S2.

##### Transfections and Luciferase Assays

HepG2 cells were cultured in DMEM medium supplemented with 10% (*v*/*v*) heat-inactivated FBS (Gibco/Invitrogen, Carlsbad, CA, USA) in a humidified atmosphere with 5% CO_2_ at 37 °C. Before transfections, HepG2 cells were seeded in 24-well cell culture plate at a density of 1.2 × 10^5^ and cultured 24 h to reach 70–80% convergence before the transient transfection. All Plasmids were transiently transfected into HepG2 cells using lipofectamine™ 2000 (Invitrogen) following the manufacture’s protocol. The 500-ng reporter plasmids were used in Opti-MEM (Invitrogen) and they were co-transfected with 25 ng pRL-TK, a Renilla luciferase reporter vector as internal control. After 4 h, the transfection medium was replaced by 10% FBS-DMEM, 10% FBS-DMEM + 0.1 μM AS and 10% FBS-DMEM + 1 μM AS. The concentration of AS was selected according to our preliminary experiments by MTT assay and relative luciferase activity of pGl3-1848/+79 of *PI3KC2b* vector assay and the results were showed in Supplementary Figure S1. MTT assay followed our previous studies [46]. After 24 h incubation, HepG2 cells were harvested to assay the luciferase activity by Dual-Luciferase Reporter Assay System (Promega) following the manufacture’s instruction. The relative luciferase activity was presented as the ratio of firefly luciferase to Renilla luciferase. All experiments were performed in triplicates and repeated at least three times.

##### Sequence Analysis

For sequence analysis of the promoters of *PI3KCa*, *PI3KC2b* and *PI3KC3* from yellow catfish, putative transcription factor binding sites were predicted by online software MatInspector (http://www.genomatix.de/), the JASPAR database (http://jaspar.genereg.net/) and the TFSEARCH database (http://www.cbrc.jp/research/db/TFSEARCH.html). Sequence alignments were assessed with the Clustal-W multiple alignment algorithm. The CpG islands were analyzed by online tool MethPrimer (http://www.urogene.org/methprimer/index1.html) with parameters window 100, shift 1, observed CpG/expected CpG ≥ 0.60 and GC % ≥ 50.

##### Site-Mutation Analysis of Several Binding Sites on the Promoters of PI3KCa, PI3KC2b and PI3KC3

To identify relevant regulatory elements within the promoter regions of PI3KCa, PI3KC2b and PI3KC3 in yellow catfish, site-directed mutagenesis was performed using Quick Change II Site-Directed Mutagenesis Kit (Vazyme, USA) according to the manufacturer’s instructions. For HNF1 mutation analysis, pGl3-360/+71of PI3KCa vectors was used as template. For FOXO1 mutation analysis, pGl3-1848/+79 of PI3KC2b vectors and pGl3-367/+59 of PI3KC3 vectors were used as templates. For IK1 mutation analysis, pGl3-1848/+79, pGl3-1522/+79, pGl3-1263/+79, pGl3-929/+79 and pGl3-623/+79 of PI3KC2b vectors were used as templates. The mutations were conducted at the positions of −304/−300 and −238/−236 for PI3KCa promoter, −1708/−1705, −1231/−1227, −1202/−1198, −947/−943 and −609/−605 for PI3KC2b promoter and −347/−344, −224/−221 and −183/−179 for PI3KC3 promoter. The mutagenesis primers were listed in Supplementary Table S3. Amplification was performed based on the following conditions: initial denaturation at 94 °C for 5 min; 30 cycles of denaturation at 94 °C for 15 s, annealing at 57 °C for 30 s and 72 °C for 1 min, followed by a final extension at 72 °C for 10 min. Then the site-mutated promoter fragments were sub-cloned to pGL3-basic vector using restriction sites Sal I and Hind III. These mutated constructs were named as followed: (1) aMut-HNF1-1, aMut-HNF1-2 and aMut-HNF1-12 of PI3KCa; (2) 2bMut1-IK1, 2bMut2-IK1, 2bMut3-IK1 and 2b-Mut4-IK1 of PI3KC2b; (3) 2bMut-FOXO1-1, 2bMut-FOXO1-2, 2bMut-FOXO1-3, 2bMut-FOXO1-4, 2bMut-FOXO1-13; 2bMut-FOXO1-14 and 2bMut-FOXO1-134 of PI3KC2b; (4) 3Mut-FOXO1-1, 3Mut-FOXO1-2, 3Mut-FOXO1-3, 3Mut-FOXO1-12, 3Mut-FOXO1-13, 3Mut-FOXO1-23 and 3Mut-FOXO1-123 of PI3KC3. All the plasmids were screened by restriction digestion and the mutations were confirmed by DNA sequencing. Then the plasmid and pRL-TK were co-transfected into HepG2 cells lines using the same method mentioned above. After 4 h, the transfection medium was replaced by 10% FBS-DMEM. After 24 h incubation, cells were harvested to assay the luciferase activity according to the procedure above.

##### Electrophoretic Mobility-Shift Assay (EMSA)

EMSA was performed to confirm the functional binding sites of HNF1, IK1 and FOXO1 according to the protocols described in our recent publications [32]. Proteins for EMSA were extracted from HepG2 cell lines. Nuclear and cytoplasmic extracts were prepared based on the method of Read et al. [53]. Protein concentrations were determined by the BCA method [54]. These extracts were stored at −20 °C until analyzed. Nuclear extracts (10 µg) were incubated 30 min at room temperature in binding buffer (20 mM HEPES, pH 7.9, 1 mM MgCl_2_, 0.5 mM DTT, 4% Ficoll, 110 mM KCl, 0.2 μg Poly(dI-dC)) prior to the addition of biotin-labeled double-stranded oligo nucleotide, as listed in Supplementary Table S4. The reaction was allowed to proceed for 30 min at room temperature before electrophoresis on 6% native polyacrylamide gels. For competitive binding studies, a 100-fold excess of unlabeled double-stranded DNA oligo with mutant binding site as listed in Supplementary Table S4, was added with the corresponding labeled one.

#### 4.2.2. Exp2. Insulin and/or AS Incubation with Hepatocytes of Yellow Catfish

Hepatocytes were isolated from yellow catfish according to our previous studies [46] and were cultured in M199 medium containing 1 mmol/L l-glutamine, 5% (*v*/*v*) FBS, penicillin (100 IU/mL) and streptomycin (100 g/mL) in a humidified atmosphere with 5% CO_2_ at 28 °C. Hepatocytes were counted using a hemocytometer based on the trypan blue exclusion method and only more than 95% cell viability were used for the present experiment. The hepatocytes were plated onto 25 cm^2^ flasks at the density of 10^6^ cells/mL. The hepatocytes then were incubated with 10 mM PBS (control), 0.1 µM AS, 1 µM AS, 0.1 µM insulin and 1 µM AS + 0.1 µM insulin. The concentration of AS was selected according to our preliminary experiments and the results were showed in Supplementary Figure S2. Each treatment was performed in triplicate and three independent experiments were carried out. AS was added 1 h prior to the addition of insulin. Hepatocyte cells were gathered for the following analysis after 48 h.

##### TG Accumulation and Enzyme Activity Analysis

Hepatocytes were homogenized in 10 mM PBS. TG was determined by the glycerol 3-phosphate oxidasep-aminophenol (GPO-PAP) method, using a commercial kit (Nanjing Jian Cheng Bio-engineering Institute, Nanjing, China) and expressed as μmol L^−1^ TG mg^−1^ cellular protein. FAS activity was determined according to the method of Chakrabarty and Leveille [55]. G6PD and 6PGD were determined by the method of Barroso et al. [56]. ME activity was assayed following Wise and Ball [57]. ICDH activity were measured according to Bernt and Bergmeyer [58]. One unit of enzyme activity (IU), defined as the amount of enzyme that converted 1μmol of substrate to product per minute at 28 °C, was expressed as U mg^−1^ soluble protein. The soluble protein concentration of homogenates was determined by the method of Bradford [59] using bovine serum albumin (BSA) as standard.

##### mRNA Level Determination by Quantitative Real-Time PCR

The mRNA levels of *PI3Ks (PI3KCa*, *PI3KC2b*, and *PI3KC3*) and genes involved in lipid metabolism (*FAS*, *G6PD*, *6PGD*, *ACCa*, *PPARγ*, *ACCb*, *PPARα*, *ATGL*, *LPL*, *CD36*, and *FABP1*) were examined by quantitative real-time PCR (Q-PCR). Q-PCR assays were performed in a quantitative thermal cycler (MyiQ™ 2 Two Color Quantitative PCR Detection System, BIO-RAD, Hercules, CA, USA) with a 20 μL reaction volume containing 10 μL SYBR Premix Ex Taq™ II (TaKaRa, Tokyo, Japan), 1 μL of diluted cDNA (10-fold), 10 mM each of forward and reverse primers 0.4 μL and 8.2 μL H_2_O. Primers are given in Supplementary Table S5. The Q-PCR parameters consisted of initial denaturation at 95 °C for 30 s, followed by 40 cycles at 95 °C for 5 s, 57 °C for 30 s and 72 °C for 30 s. All reactions were performed in duplicates and each reaction was verified to contain a single product of the correct size by agarose gel electrophoresis. A non-template control and dissociation curve were performed to ensure that only one PCR product was amplified and that stock solutions were not contaminated. A set of seven common housekeeping genes (*β-actin*, *18s-rRNA*, *GAPDH*, *RPL7*, *HPRT*, *UBCE* and *TUBA*) were selected in order to test their transcription stability. Two most stable control genes (*GAPDH*, *18s-rRNA*, M = 0.35) were selected by using geNorm software [60]. The relative expression levels were calculated with the “delta–delta Ct” method [61] when normalizing to the geometric mean of the best combination of two genes as suggested by geNorm. 2.7. The similar protocols have been used in other studies [62,63].

#### 4.2.3. Statistical Analysis

Statistical analysis was performed with SPSS 19.0 software. Results are presented as means ± SEM. For assay of promoters’ activities, student’s *t*-test is used to determine the differences between the two groups. For the insulin and AS incubation experiment, the homogeneity of variances among the treatments was tested using Bartlett’s test, they were then subjected to one-way ANOVA followed by Tukey’s multiple range test. Difference was considered significant at *p* < 0.05.

## Supplementary Materials

The following are available online at www.mdpi.com/1422-0067/19/1/265/s1.

## Abbreviations

6PGD	6-phosphogluconate dehydrogenase
ACC	acetyl-CoA carboxylase
Akt	protein kinase B
AP1	activator protein-1
AS	AS1842856
ATGL	adipose triacylglyceride lipase
BRE	B recognition element
CD36	fatty acid translocase
CPT IA	carnitine palmitoyltransferase IA
DCE	downstream core element
DRE	DNA recognition element
eEF	translation elongation factor
FAS	fatty acid synthase
FABP1	fatty acid binding protein 1
FBP	fatty acid binding protein
FOXO1	forkhead box O
G6PD	glucose 6-phosphate dehydrogenase
GAPDH	glyceraldehyde-3-phosphate dehydrogenase
HNF1	hepatocyte nuclear factor 1
HPRT	hypoxanthineguanine phosphoribosyltransferase
ICDH	isocitrate dehydrogenase
IK1	ikaros zinc finger 1
LPL	lipoprteinlipase
ME	malic enzyme
MS-222	tricaine methanesulfonate
MTT	3-(4,5-dimethylthiazol-2-yl)-2,5-diphenyltetrazoliumbromide
NF-κB	nuclear factor-kappa B
PBS	phosphate-buffered saline
PI3K	Phosphatidylinositol-3 kinase
PPAR	peroxisome proliferators-activated receptors
RPL7	ribosomal protein L7
STAT	Signal transducers and activators of transcription protein
TFBS	transcription factor binding site
TG	triacylglyceride
TUBA	tubulin α chain
UBCE	ubiquitin-conjugating enzyme
