# Supplementary material for: Functional Analysis of Promoters from Three Subtypes of the PI3K Family and Their Roles in the Regulation of Lipid Metabolism by Insulin in Yellow Catfish Pelteobagrus fulvidraco
Source: Int J Mol Sci. 2018 Jan 16;19(1):265. doi: 10.3390/ijms19010265 (PMC5796211; doi:10.3390/ijms19010265)
Supplement: Supplementary file 1 [file ijms-19-00265-s001.pdf]

Table 1. Primers used for PI3KCa, PI3KC2b, and PI3KC3 promoter cloning

| Gene | Forward primer (5′-3′) | Reverse primer (5′-3′) | Step |
|---|---|---|---|
| *PI3KCa* | GTCTGTCGTTGCTATGGAGAGC | CCATAGAGCAGTTTCAGGGTA | RT-PCR |
| | ACGATGGACTCCAGAGCGGCCGCVVNVNNNCCAA | GAGTGTCCAAGCGAAATAGTAAATACG | Hitail-PCR1 |
| | ACGATGGACTCCAGAG | TTTCTGTTGAGTAATCCGCTCTCGTG | Hitail-PCR2 |
| | ACGATGGACTCCAGAG | CATGGTCGTTTAATCCAGGAATGAAGA | Hitail-PCR3 |
| *PI3KC2b* | CGCTGAACTGAGAATCTCGA | TTGATCTGCGGTCCTTATGA | RT-PCR |
| | ACGATGGACTCCAGAGCGGCCGCBDNBNNNCGGT | ATCACACATGGGTTCTAACACACTGC | Hitail-PCR1 |
| | ACGATGGACTCCAGAG | ACGACGCTTAAATTCCGCACTGTATA | Hitail-PCR2 |
| | ACGATGGACTCCAGAG | GAAGGTTTTCGGTTTGTTGCTTTCAG | Hitail-PCR3 |
| *PI3KC3* | ATCGATTGCTTATGCCTGTT | CCAGGTCGCAACTGTAAACAT | RT-PCR |
| | ACGATGGACTCCAGAGCGGCCGCBNBNNNGGTT | GCAGATATTCACTCAGACTTTGACACA | Hitail-PCR1 |
| | ACGATGGACTCCAGAG | CACGGTGATTTTATACGGTGTGTGACTTTA | Hitail-PCR2 |
| | ACGATGGACTCCAGAG | ACAACTGACTGGAGATGGACACGGAC | Hitail-PCR3 |

Table 2. Primers used for 5’-deletion plasmids construction

| Primers | Forward primer (5′-3′) | Reverse primer (5′-3′) |
|---|---|---|
| *PI3KCa* | | |
| pGl-360/+71 | ctatcgataggtaccgagctcGCAATCTAAGCAATTTAT | cagtaccggaatgccaagGCTTGATGGAGACGCTA |
| pGl-224/+71 | ctatcgataggtaccgagctcAAAAGGAATCTGGATGC | cagtaccggaatgccaagGCTTGATGGAGACGCTA |
| pGl-86/+71 | ctatcgataggtaccgagctcATTTGTGTCGCATTACG | cagtaccggaatgccaagGCTTGATGGAGACGCTA |
| *PI3KC2b* | | |
| pGl-1848/+79 | ctatcgataggtaccgagctcGGCAGTTGAGCCAGCA | cagtaccggaatgccaagcttTCGCAGAAGGTTTTCG |
| pGl-1522/+79 | ctatcgataggtaccgagctcCTGC | cagtaccggaatgccaagcttTCGCAG |

| | | |
|---|---|---|
| | CGGTGGTCTTCTTCCA | AAGGTTTTCG |
| pGl-1263/+79 | ctatcgataggtaccgagctcCATG GATTCAAATCCTT | cagtaccggaatgccaagcttTCGCAG AAGGTTTTCG |
| pGl-929/+79 | ctatcgataggtaccgagctcGCAC TACCATGCACTTCTC | cagtaccggaatgccaagcttTCGCAG AAGGTTTTCG |
| pGl-623/+79 | ctatcgataggtaccgagctcTCCCC AGATGAAGTTCCC | cagtaccggaatgccaagcttTCGCAG AAGGTTTTCG |
| pGl-388/+79 | ctatcgataggtaccgagctcAGGG TTTCACAATATCG | cagtaccggaatgccaagcttTCGCAG AAGGTTTTCG |
| pGl-144/+79 | ctatcgataggtaccgagctcCACG ACAAAAGAAAGCATC | cagtaccggaatgccaagcttTCGCAG AAGGTTTTCG |
| pGl-94/+79 | ctatcgataggtaccgagctcAAAT CTCTGATCCGAGC | cagtaccggaatgccaagcttTCGCAG AAGGTTTTCG |
| *PI3KC3* | | |
| pGl-367/+59 | ctatcgataggtaccgagctcTGGT GCTATGTGTTAAAG | cagtaccggaatgccaagCATAGTGC GTTTCCCGA |
| pGl-291/+59 | ctatcgataggtaccgagctcCATA CTTTCACATGAAAAGC | cagtaccggaatgccaagCATAGTGC GTTTCCCGA |
| pGl-85/+59 | ctatcgataggtaccgagctcGCGG CTCTAAATCTCT | cagtaccggaatgccaagCATAGTGC GTTTCCCGA |

Table.3 Primers used for site-mutation analysis

| Primers | Forward primer (5′-3′) | Reverse primer (5′-3′) |
|---|---|---|
| aMut-HNF1-1 | TAAAAGgggggAAAAAAATTT ATATCAGATCCAGAGTTTG | TTTTTTcccccCTTTTAATAAA AAAAAGAAAACTTATATTC AAG |
| aMut-HNF1-2 | AATTATGgggAAGCTCCATTA AAAGGAATCTGGA | GGAGCTTcccCATAATTCATA GTCTGACATTATGACTGAA |
| 2bMut-FOXO1-1 | TTTGTGggggCACACAAACAG GCTCACAAATGC | TTTGTGTGccccCACAAACA GTGACCAGTTGCGC |
| 2bMut-FOXO1-2 | ATTCAGTAgggggAAGCCATGG TTCACAGCCAA | GCTTcccccTACTGAATTTAC GGAAGGATTTGAAT |
| 2bMut-FOXO1-3 | AAACTGgggggACTTCGACAT GCCAAGGAAGAT | CGAAGTcccccCAGTTTGGCT GTGAACCATGG |
| 2bMut-FOXO1-4 | TTGTACgggggCAATACTGTTA TTTGCACTACCATGC | GTATTGcccccGTACAATATG CAGCAGCAGCAGT |
| 2bMut-IK1 | ATGAAggggggATGGAAAGTTT CCGGCCCCTT | TTCCATccccccTTCATCTGGG GAGAGCTCGG |
| 3Mut-FOXO1-1 | AAAGTAggggAGAAAACCTTT AAAAATTCTAAGCTAAGAT | GGTTTTCTccccTACTTTAAC ACATAGCACCAGAGCTCG |
| 3Mut-FOXO1-2 | GACTTATTAGATggggTATTAA TTCAAATACAATTAAATAATA TGAATAATAAA | TAccccATCTAATAAGTCATA GTTTAAACTAGAATGTGTT G |

| | | |
|---|---|---|
| 3Mut-FOXO1-3 | TAAgggggACATAAAGGACTGAGACGGGGTGA | CCTTTATGTcccccTTATTATTCATATTATTTAATTGTATTTGAATTAATAA |

Table 4 Primers used for electrophoretic mobility-shift assay

| Primers | Forward primer (5′-3′) | Reverse primer (5′-3′) |
|---|---|---|
| B-PI3KCa-HNF1 | TATGAATTATGTTAAAGCTCCA | TGGAGCTTTAACATAATTCATA |
| Mut-PI3KCa-HNF1 | TATGGGGTATGTTAAAGCTCCA | TGGAGCTTTAACATACCCCATA |
| B-PI3KC2b-FOXO1-1 | CACTGTTTGTGAACACACACAA | TTGTGTGTGTTCACAAACAGTG |
| Mut-PI3KC2b-FOXO1-1 | CACTGTTTGTGGGGGCACACAA | TTGTGTGCCCCCACAAACAGTG |
| B-PI3KC2b-FOXO1-2 | GCCAAACTGAAAAAACTTCGAC | GTCGAAGTTTTTTCAGTTTGGC |
| Mut-PI3KC2b-FOXO1-2 | GCCAAACTGGGGGGACTTCGAC | GTCGAAGTCCCCCCAGTTTGGC |
| B-PI3KC2b-FOXO1-3 | TGTACAAAAACAATACTGTTAT | ATAACAGTATTGTTTTTGTACA |
| Mut-PI3KC2b-FOXO1-3 | TGTACGGGGGCAATACTGTTAT | ATAACAGTATTGCCCCCGTACA |
| B-PI3KC2b-IK1 | CAGATGAAGTTCCCATGGAA AG | CTTTCCATGGGAACTTCATCTG |
| Mut-PI3KC2b-IK1 | CAGATGAAGGGGGGATGGAA AG | CTTTCCATCCCCCCTTCATCTG |
| B-PI3KC3-FOXO1 | TATTAGATTGTTTATTAATTCA | TGAATTAATAAACAATCTAATA |
| Mut-PI3KC3-FOXO1 | TATTAGATGGGGTATTAATTCA | TGAATTAATACCCCATCTAATA |

Table 5. Primers used for q-PCR from yellow catfish

| Primers | Forward primer (5′-3′) | Reverse primer (5′-3′) |
|---|---|---|
| β-Actin | GGACTCTGGTGATGGTGTGA | CTGTAGCCTCTCTCGGTCAG |
| 18srRNA | CTGCCGGTGGTCTTCTTCCA | ATTCAGCGGGTCGTCTCGTC |
| RPL17 | GGCAAATGTACAGGAGCGAG | GCCTTGTTGAGCTTGACGAA |
| GAPDH | TTTCAGCGAGAGAGACCCAG | ATGACTCTCTTGGCACCTCC |
| HPRT | ATGCTTCTGACCTGGAACGT | TTGCGGTTCAGTGCTTTGAT |

| | | |
|---|---|---|
| UBCE | TCAAGAAGAGCCAGTGGAGG | TAGGGGTAGTCGATGGGGAA |
| TUBA | TCAAAGCTGGAGTTCTCGGT | AATGGCCTCGTTATCCACCA |
| PI3KCa | CCAGGGAAACGGGATACA | TCCACTTTACAGCAAGGAGAA |
| PI3KCb | CTGATGCTGACTTCTGGGTTA | TTATGGAGGTGTTAGGAGGGA |
| PI3KCg | CCGATTTTGTTCTGAGGGT | TTCTCCACTTTTCAGGCACT |
| PI3KCd | GACACCCAAAAGAACCACG | TTCCCACATTGAGCCACC |
| PI3KC2a | TGAATCAGAACCCAAGAAGAAG | GGATGCGAAATGTAACGGA |
| PI3KC2b | GCGGAATTTAAGCGTCGTC | GGGGATTTGGAGGGATAAGA |
| PI3KC3 | AAAGCAGGCACTGGAGTTATT | GCGTAGCGTCTGACTGTAGG |
| FAS | AACTAAAGGCTGCTGGTTGCTA | CACCTTCCCGTCACAAACCTC |
| G6PD | CAGGAATGAACGCTGGGATG | TCTGCTACGGTAGGTCAGGTCC |
| 6PGD | GCTCTGATGTGGCGAGGTGG | CGTAGAAGGACAGTGCAGTGGTAAA |
| CPT IA | ATTTGAAGAAGCACCCAGAGTATGT | CCCTTTTATGGACGGAGACAGA |
| PPARα | CGAGGATGGGATGCTGGTG | CGTCTGGGTGGTTCGTCTGC |
| PPARγ | ACGCCCCGTTCGTTATCC | TGAGCAGAGTCACCTGGTCATTG |
| ACCa | AATGGCTATGACTGGCAAGGG | TACCGTCGTTCCACTAGTGATTT |
| ACCb | AAGTGCTGTCTGAGCCCTGGAG | CGCGGATCCTCCACTAGTGATTTCACTATAGG |
| ATGL | TTGCGGAAATGTGATTGAGGT | CACGGAAGGCAGGAGGGA |
| LPL | AGCGATTGGTGGGAGGATTAT | TGAGCACGGTCCAGTTTCCT |
| CD36 | GATCGTTCTGATTTCGGTTGG | TTATTGTCGTACTTCGGCACTG |
| FABP | GAAAGTGGACCGCAATGAGA | GTCAGTGTTTTGCCGTTGTCTT |

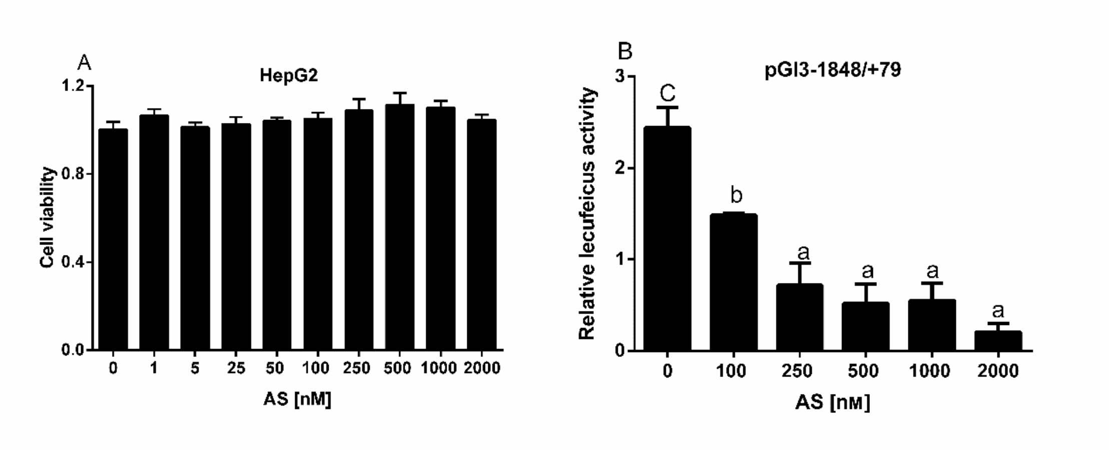


Fig.1. (A) Effects of AS1842856 (AS) on cell viability on HepG$_2$ cells, and no significant differences were found on the cell viability after incubation for24 h. (B) Effects of AS on the relative luciferase activity of pGl3-1848/+79 of PI3KC2b-vectors in HepG$_2$ cells. Values are expressed as means+s.e.m. (N=3). Different letters indicate significant differences among groups ($P<0.05$).

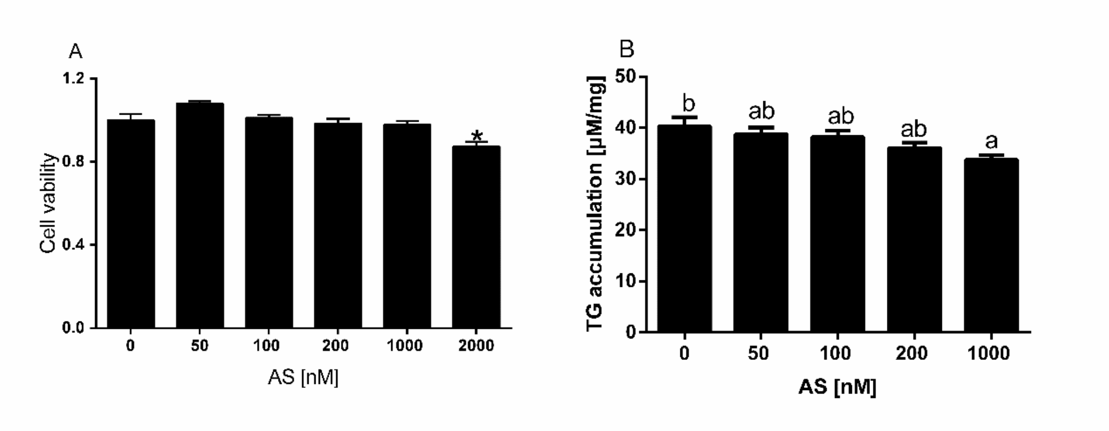


Fig.2 (A) Effects of AS on cell viability on primary hepatocytes from yellow catfish after incubation for 48 h. (B) Effects of AS on triglyceride content in primary hepatocytes from yellow catfish. Symbol (*) indicates significant differences between the treatment group and the control group ($P<0.05$).
